# Nipple Preserving Wise-Pattern Mastopexy Following Deep Inferior Epigastric Perforator Flap Breast Reconstruction: Description of the Surgical Technique and Clinical Results

**DOI:** 10.1016/j.jpra.2024.12.001

**Published:** 2024-12-05

**Authors:** Osama Darras, Sara Yacoub, Diwakar Phuyal, Raffi Gurunian, Sarah N. Bishop

**Affiliations:** Department of Plastic Surgery, Cleveland Clinic, Ohio, USA

**Keywords:** Breast, Breast corrective surgery, Mastopexy, Free flap

## Abstract

Breast revision surgery is often necessary in patients following postmastectomy breast reconstruction with free autologous flaps for aesthetic improvement. Indications for nipple-sparing mastectomy continue to be expanded oncologically. However, revision techniques for aesthetic concerns following breast reconstruction are underreported in the literature. Therefore, we describe a mastopexy technique following deep inferior epigastric perforator (DIEP) flap breast reconstruction after nipple-sparing mastectomy to correct ptosis and reshape the breast. The blood supply of the nipple-areolar-complex is through the microvasculature of the DIEP flap and subdermal plexus. We report three patients who underwent nipple preserving Wise-pattern mastopexy following DIEP flap breast reconstruction.

## Introduction

Breast cancer is one of the most common malignancies affecting women worldwide, with mastectomy being widely used as treatment. Advances in reconstructive surgery have significantly improved the quality of life for patients undergoing mastectomy.^1^ The deep inferior epigastric perforator (DIEP) flap is commonly used for breast reconstruction due to its well-documented long-term benefits.^2^^,^^3^ Additional revision procedures are commonly required to address residual aesthetic concerns^4^^,^^5^ that include breast asymmetry, removal of the DIEP flap skin paddle, scar revision, improvement of the shape, ptosis of the breasts, and nipple-areolar-complex (NAC) malposition. There is an opportunity with nipple-sparing mastectomies (NSM) undergoing DIEP flap reconstruction to create an aesthetically reconstructed breast resembling breasts following cosmetic surgeries. This drives surgeons to innovate and refine surgical techniques to fulfill patient's demands.

Studies have documented various corrective procedures following DIEP flap reconstruction, such as flap revision for reshaping, nipple reconstruction, implant insertion, and vessel exploration.^6^^,^^7^ Flap contouring revisions were reported to be the most performed additional operative procedure, often followed by breast fat grafting and contralateral mastopexy for symmetry.^8^ Despite the frequency of these revisions,^9^ there is a lack of detailed description of the surgical technique for contouring the breast post-DIEP reconstruction and of a nipple preserving Wise-pattern mastopexy after DIEP flap reconstructions that mainly derives the blood supply from the DIEP flap microvasculature.

We describe the surgical technique of the DIEP Wise-pattern mastopexy (DWPM) in three patients who have undergone NSM and DIEP flap breast reconstruction to achieve satisfactory size and shape along with repositioning of the NAC. This technique was reported by DellaCroce et al.^10^ Our enhancements to this technique include the use of indocyanine green (ICG) angiography for NAC perfusion. We explored different uses for this technique and the role of radiation and previous periareolar surgeries. This paper addresses techniques to salvage the NAC when there are concerns about NAC ischemia.

## Surgical Technique

The surgical technique involves the adaptation of the Wise-pattern drawing, which aims to address aesthetic concerns by reshaping the breast and repositioning the NAC.

Patients who have ptosis and desire to have a smaller breast size after DIEP flap reconstruction are ideal patient candidates for this technique. The Wise-pattern marking^11^ is performed on the patient's skin, similar to conventional breast reduction markings. Sternal notch to nipple distance is personalized depending on the patient's physical appearance. A 42 mm cookie cutter is used to mark the NAC, and the entire Wise-pattern is completely de-epithelialized.

The next step involves careful dissection of the mastectomy skin flap superiorly, mimicking the technique used in inferior pedicle breast reduction surgery. This dissection severs the NAC from its superior attachments while preserving perfusion via the DIEP flap and the inferior dermal mastectomy flap (Figure 1.A). Particular attention is given to the medial aspect of the breast due to the proximity of the DIEP pedicle as all recipient vessels were the internal mammary vessels. In cases requiring debulking, excess fat can be safely excised from the lateral and superior DIEP flap.

**Figure 1.A:**
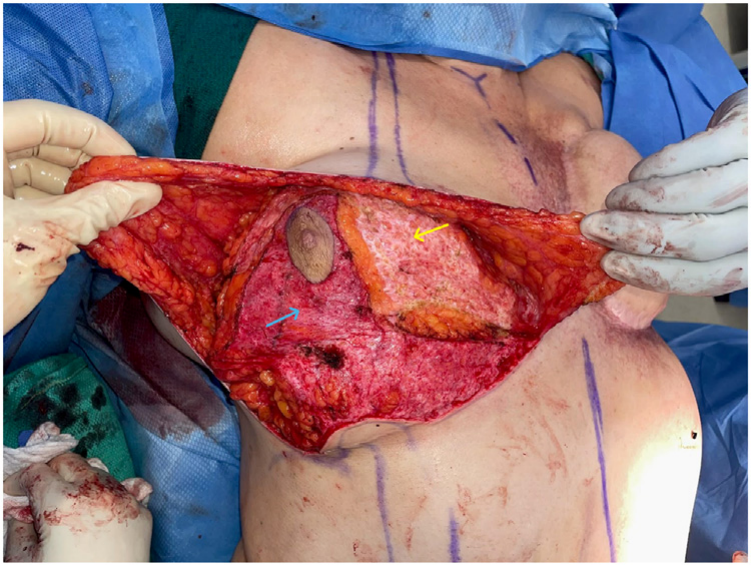
Intraoperative view after de-epithelization of the Wise-pattern and raising superior mastectomy flaps. The yellow arrow indicates the DIEP flap and blue arrow indicates the de-epithelialized mastectomy flap connecting to the DIEP flap.

The patient is then seated upright to facilitate accurate marking of the new NAC inset using a 38 mm cookie cutter. After marking, the patient is repositioned supine, and the new NAC location is excised.

The perfusion of the NAC is confirmed using ICG angiography. In case of marked concerns about NAC perfusion, nitroglycerin can be placed, or ultimately, a free nipple graft can be considered (Figure 1.B).

**Figure 1.B:**
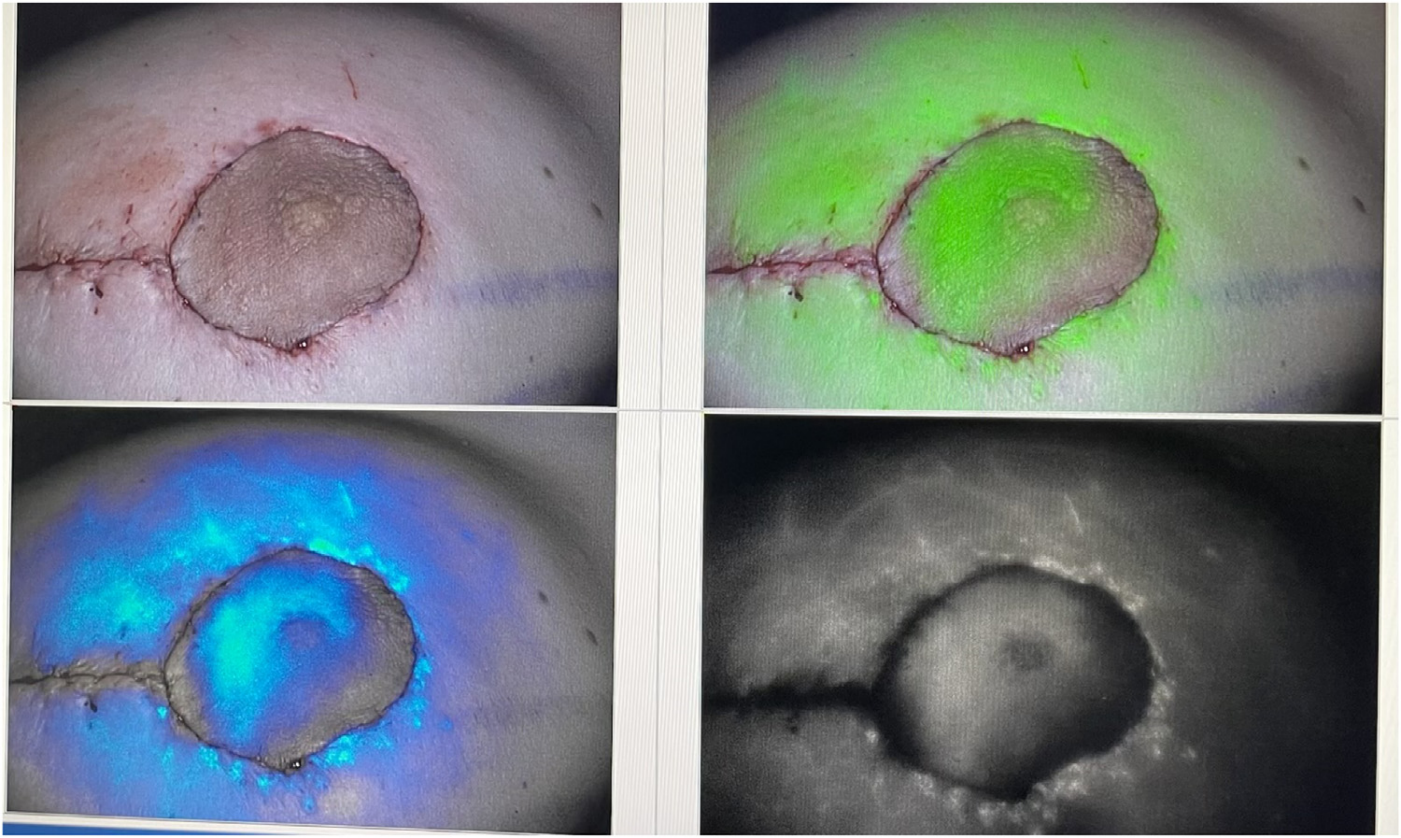
Intraoperative view after injecting the ICG, which shows the perfusion of the NAC.

## Cases

### Patient #1

**Figure 2:**
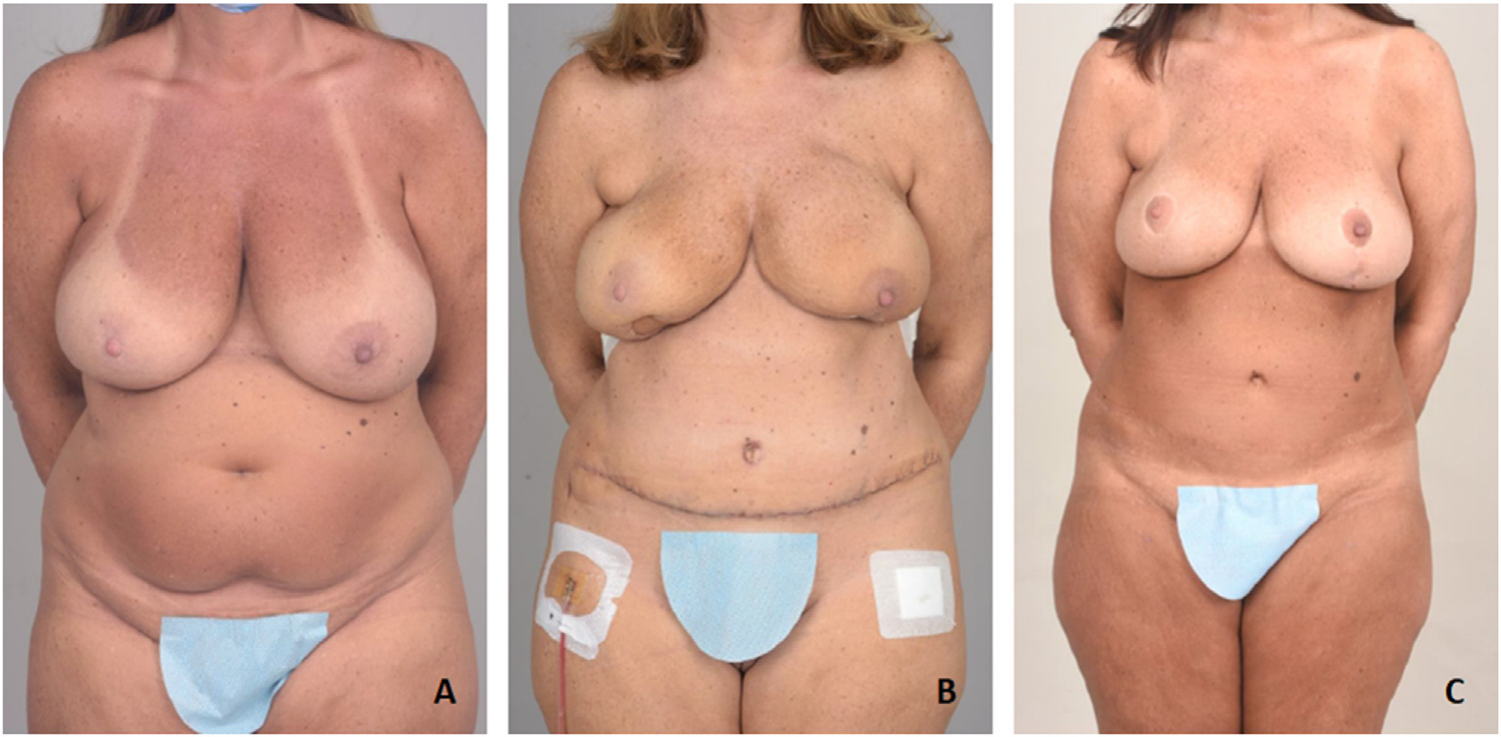
A: Preoperative photo. B: Postoperative photo 16 days after NSM and immediate DIEP flap breast reconstruction. C: Final result. One year after DWPM.

A 48-year-old female patient (Figure 2.A) underwent bilateral NSM with immediate bilateral DIEP flap reconstruction due to right-side breast cancer (Figure 2.B). She complained of large and ptotic breasts and requested surgical intervention. Five months after immediate DIEP reconstruction, she underwent bilateral Wise-pattern mastopexy and reduction of her breast reconstruction. Intraoperatively, ICG angiography showed excellent perfusion in the left NAC but some perfusion deficit superiorly in the right NAC, which led to the use of nitroglycerin ointment on the right nipple only. Nitroglycerin ointment was placed only once and not continued. Her postoperative course was unremarkable. She underwent further fat grafting to the breast for contour correction. The patient is content with her aesthetic results. Figure 2.C shows the patient's final result after more than one year of undergoing DWPM.

### Patient #2

**Figure 3:**
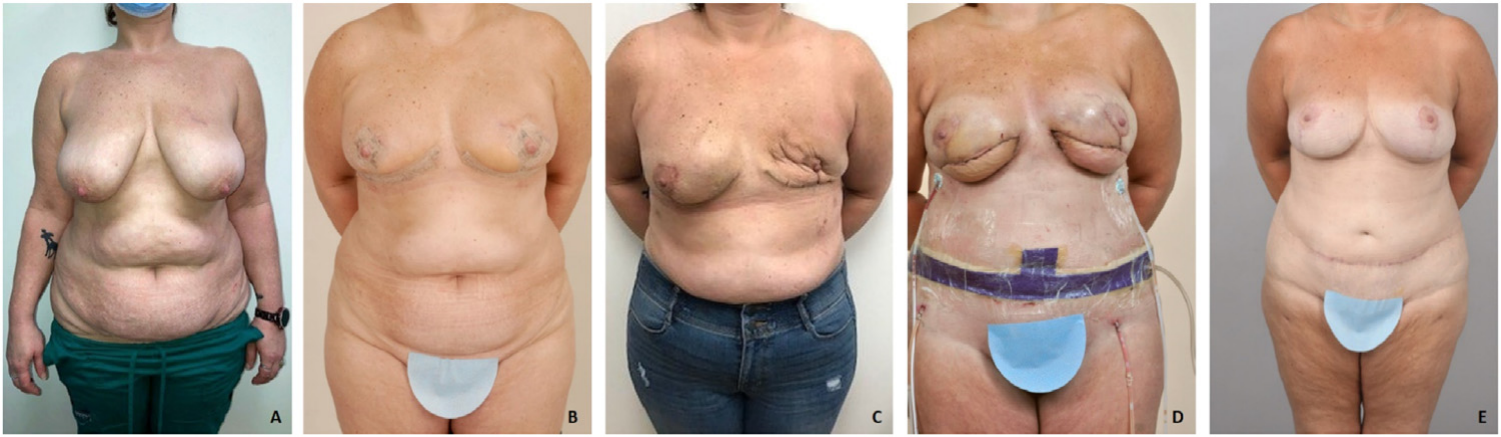
A: Preoperative photo. B: Postoperative photo 10 days after superomedial breast reduction. C: Postoperative photo 1 month after NSM and left tissue expander removal due to infection. D: Postoperative photo 9 days after bilateral DIEP reconstruction. E: Final result 18 months after DWPM.

A 42-year-old (Figure 3.A) female with a BRCA1 gene underwent bilateral, superomedial-pedicled staged breast reduction surgery to decrease ptosis and enable a NSM (Figure 3.B). The patient then underwent bilateral prophylactic NSM and immediate breast reconstruction with prepectoral tissue expanders with acellular dermal matrix (ADM) complicated by left breast tissue expander infection for which she had removal of the tissue expander and ADM (Figure 3.C). Three months later, she underwent bilateral DIEP flaps (Figure 3.D); despite breast reduction to correct ptosis, she still had inadequate NAC position after DIEP flap reconstruction. We decided that the best procedure to improve her NAC position shape and remove the DIEP flap skin paddle was a DWPM (Figure 3.E.) The Wise-pattern mastopexy enabled not only breast reshaping but also the removal of an area of fat necrosis on the left breast.

### Patient #3

**Figure 4:**
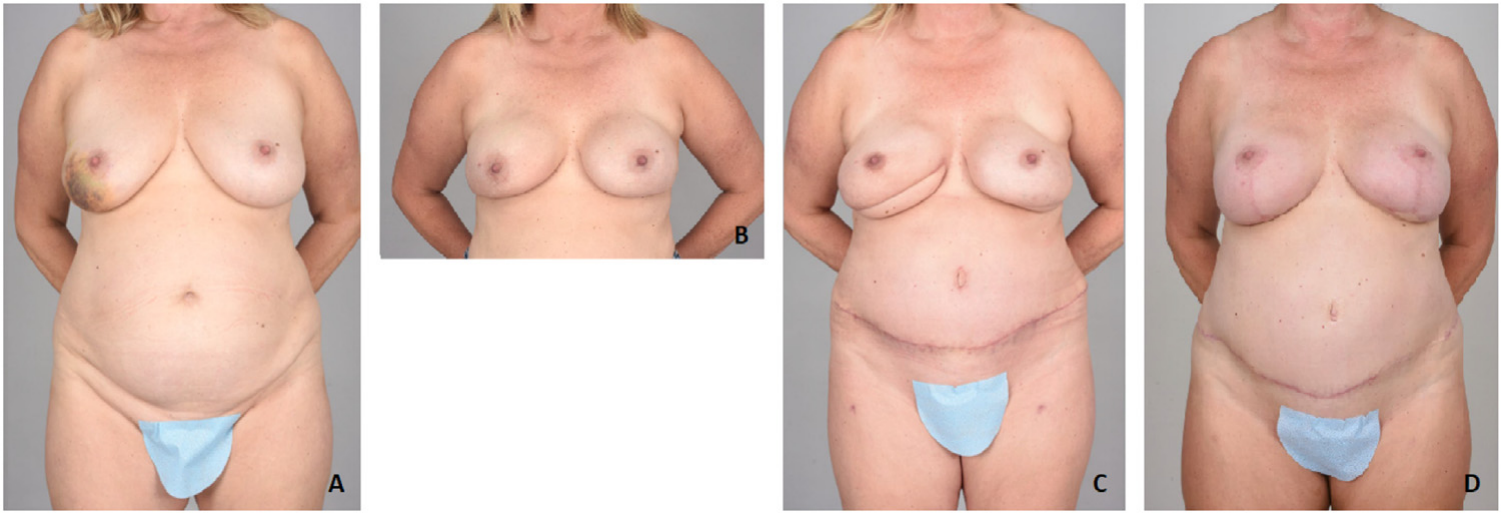
A: Preoperative photo. B: Postoperative photo 2 months after bilateral NSM and tissue expander insertion. C: Postoperative photo 2 months after bilateral DIEP flap breast reconstruction. D: Four months after DWPM.

A 56-year-old female (Figure 4.A) with right breast cancer underwent bilateral NSM. Immediate reconstruction included bilateral breast reconstruction with placement of prepectoral tissue expanders with ADM. (Figure 4.B). Following the course of treatment 3 months later, the patient underwent bilateral DIEP flap breast reconstruction. The postoperative course was uncomplicated (Figure 4.C). Seventeen months later, the patient underwent bilateral DWPM. Intraoperatively, ICG angiography was used to assess NAC perfusion without concern. The postoperative course was unremarkable. Aesthetic result of the patient 4 months postoperatively is shown in Figure 4.D.

## Discussion

The DWPM is a helpful technique following DIEP flap breast reconstruction and NSM to correct persistent ptosis, reposition the NAC, debulk the breast volume, and remove the DIEP flap skin paddle (also known as monitoring island), and any fat necrosis. Our case report highlights the practical application and benefits of DWPM, addressing the need for aesthetic improvements in patients who have undergone these surgical procedures.

The primary objective of DWPM is to enhance the aesthetic outcome for patients experiencing asymmetry, an unsightly DIEP flap skin paddle, or mispositioned NAC. Our cases demonstrated that DWPM effectively addresses these concerns by reshaping the breast and repositioning the NAC, leading to a more natural and symmetrical appearance. The ability to correct breast contour divots through fat grafting, coupled with the precise excision of excess skin and scar tissue, underscores the technique's versatility and effectiveness. This technique also succeeded in staged breast reduction,^12^^,^^13^ followed by NSM.

A critical aspect of DWPM is the preservation of the NAC perfusion. In NSM, the NAC's blood supply is derived from the mastectomy skin flap. The nipple-sparring approach was either an inframammary fold approach or L-pattern incision with the vertical limb carried from the NAC to the inframammary fold and then extended medially. The latter approach might have severed the inferior blood supply to the NAC from the mastectomy skin flap, highlighting the importance of the blood supply from the DIEP flap. Some of our patients underwent delayed DIEPs with the removal of tissue expanders, in which we removed all of the capsules surrounding the tissue expanders during the DIEP flap to ensure proper integration of the DIEP flap to the mastectomy flap. During the DWPM, after the entire Wise-pattern is de-epithelialized, the superior mastectomy flaps are raised, divorcing the NAC from the superior mastectomy flaps. The NAC now mainly receives blood supply through its connections to the DIEP flap microvasculature. DIEP flap promotes the development of a robust microvascular network, enhancing NAC perfusion. Exploring what other surgeons did, Zafar and Ellsworth^14^ showed survival to skin paddles with similar mastopexy techniques after DIEP flap reconstruction with beautiful results. However, they did not present the same technique of preserving the native NAC. Zafar and Ellsworth^14^ advocate waiting 3 months after DIEP flap to allow for microvasculature formation. Similarly, we recommend at least 3-6 months after the DIEP flap to perform revisions. Also, it is important to note that the connections of the NAC to the DIEP flap are tenuous and one needs to exercise gentle technique and tissue handling to not disrupt the blood supply. Antoniazzi et al^15^ described a NSM technique combined with Wise-pattern mastopexy with a tissue expander or an implant where they de-epithelialized the periareolar mastectomy flap with the inferior pedicle. The dermal mastectomy flap was preserved all around the NAC, which shows the difference between our technique and this technique. However, it highlights the role of the dermal mastectomy flap in supplying the NAC. On the contrary, in our technique, one of the reported mastectomy techniques was an L-shaped incision, which severed the inferior mastectomy dermal flap, showing the importance of the blood supply from the NAC and of the delay between the mastectomy and the mastopexy, which allows the tissue to recover from previous undermining of tissue supply.

ICG fluorescence imaging plays a crucial role in the intraoperative assessment of NAC perfusion.^16^, ^17^, ^18^ Our findings suggest that ICG angiography is a reliable method for evaluating and ensuring adequate blood flow to the NAC, thereby reducing the risk of ischemic complications by performing an immediate intervention to salvage the NAC. The use of nitroglycerin ointment in cases of low perfusion further exemplifies the proactive measures that can be taken to mitigate the risk of necrosis. Nitroglycerin ointment was reported to significantly decrease the rate of skin flap necrosis after NSM.^19^ Our practice is to place nitroglycerine operatively one time when vascular malperfusion concerns arise. Postoperative use is usually not done to ameliorate the risks of side effects, including headaches and patient discomfort. Ultimately, we would perform a free nipple graft if there was marked concern for nipple malperfusion, which is discussed preoperatively with the patient.

## Conclusion

DWPM following DIEP flap breast reconstruction after NSM is a corrective technique that can be performed for patients complaining of ptotic breasts after DIEP flap breast reconstruction. Our findings demonstrate that DWPM can improve aesthetic outcomes while maintaining NAC perfusion.
